# Occult gram-negative bacterial colonization of explanted alloplastic implants incidentally detected during revision rhinoplasty: a microbiologically confirmed case report

**DOI:** 10.1097/RC9.0000000000000986

**Published:** 2026-09-11

**Authors:** Phung Nguyen Van, Cuu Nguyen Cong

**Affiliations:** a Department of Plastic Surgery, School of Medicine, Tra Vinh University, Tra Vinh, Vietnam; b The Gioi Dep Aesthetic Surgery Clinic, Ho Chi Minh City, Vietnam

**Keywords:** biofilm, case report, Enterobacter aerogenes, explanted alloplastic implant, latent bacterial colonization, revision rhinoplasty

## Abstract

**Background:**

Postoperative infection following rhinoplasty is uncommon but can have serious consequences, particularly after revision procedures. Latent bacterial colonization and biofilm formation on implanted materials may contribute to delayed or subclinical infection, yet microbiologically confirmed colonization of implants in fully asymptomatic patients is rarely documented, and Gram-negative organisms are seldom reported in this setting.

**Case presentation:**

A 35-year-old woman underwent revision rhinoplasty for a persistent nasal deformity after ten prior procedures involving alloplastic materials and autologous cartilage. She had no clinical signs of infection and had normal laboratory findings. Previously implanted expanded polytetrafluoroethylene (ePTFE) and residual cartilage grafts were explanted, and intraoperative nasal fluid from the implant pocket was cultured, yielding *Enterobacter aerogenes* with an AmpC-type phenotype (resistant to third-generation cephalosporins and susceptible to cefepime, carbapenems, aminoglycosides, and fluoroquinolones). Because viable Gram-negative organisms were confirmed adjacent to a newly placed autologous costal cartilage framework, a targeted 7-day course of meropenem and amikacin was given according to susceptibility testing to protect the reconstruction. At the 3-month follow-up, the surgical site was stable with no evidence of infection.

**Discussion:**

Viable bacteria may persist on implanted materials without clinical manifestations, consistent with biofilm formation. As no clinical, laboratory, or intraoperative signs of infection were present, the finding is best characterized as occult (latent) colonization rather than active infection and may be overlooked during revision rhinoplasty.

**Conclusion:**

Latent bacterial colonization of previously implanted materials in revision rhinoplasty may be under-recognized. Routine microbiological evaluation of explanted materials may facilitate early detection and guide postoperative management.

## Introduction

Augmentation rhinoplasty with alloplastic implants – commonly silicone, porous polyethylene (Medpor), or expanded polytetrafluoroethylene (ePTFE) – is among the most frequently performed facial esthetic procedures. Although generally considered safe, complications such as implant displacement, extrusion, capsular contracture, and infection may still occur.

Bacterial biofilms are increasingly implicated in implant-related infections, allowing organisms to adhere to implant surfaces and resist both antibiotic therapy and host immune responses[1]. Relatedly, occult bacterial colonization – persistence of organisms on implants without overt inflammatory signs – is recognized in esthetic surgery[2], but microbiologically confirmed occult colonization in asymptomatic revision-rhinoplasty patients is rarely described.

HIGHLIGHTS

- Occult Gram-negative colonization was detected in an asymptomatic revision rhinoplasty.
- *Enterobacter aerogenes* was isolated from an explanted ePTFE implant pocket.
- There were no clinical, laboratory, or intraoperative signs of infection despite a positive culture.
- Findings support latent colonization and biofilm formation on retained implants.
- Routine culture of explanted materials may aid in early detection and graft protection.

We report *Enterobacter aerogenes* recovered from an ePTFE implant during revision rhinoplasty in a patient without any preoperative signs of infection. The case is notable for three reasons: detection in the complete absence of clinical, laboratory, and intraoperative signs of infection, illustrating truly occult colonization; a Gram-negative causative organism atypical for rhinoplasty, where Gram-positive commensals predominate and are infrequently documented on nasal implants; and incidental recovery from explanted ePTFE, highlighting the implant as a durable bacterial reservoir and arguing for routine microbiological sampling of explanted material even when infection is not clinically suspected. This work is reported in line with the SCARE 2025 guidelines[3].

## Case presentation

A 35-year-old woman was referred for revision rhinoplasty. She had no significant medical history, took no regular medication, had no known drug allergies or relevant family history, and denied smoking or alcohol use. Her initial rhinoplasty had been performed for cosmetic dorsal augmentation with a silicone implant, followed by nine further procedures (10 in total): (1) primary silicone augmentation; (2) removal and replacement of the silicone implant for an unsatisfactory outcome; (3) ePTFE implant with auricular cartilage; (4) ePTFE removal after a postoperative infection; (5) ePTFE implant with auricular cartilage; (6) costal cartilage with an ePTFE implant; (7) ePTFE removal after a second postoperative infection, with costal cartilage preserved; (8) ePTFE reinsertion with auricular-cartilage tip coverage; (9) inguinal fat graft for nasal-tip contracture with implant exposure; and (10) gluteal fat grafting for persistent contracture and tip exposure (Fig. 1). Her history was thus notable for two prior postoperative infections and recurrent implant exposure.

**Figure 1.**
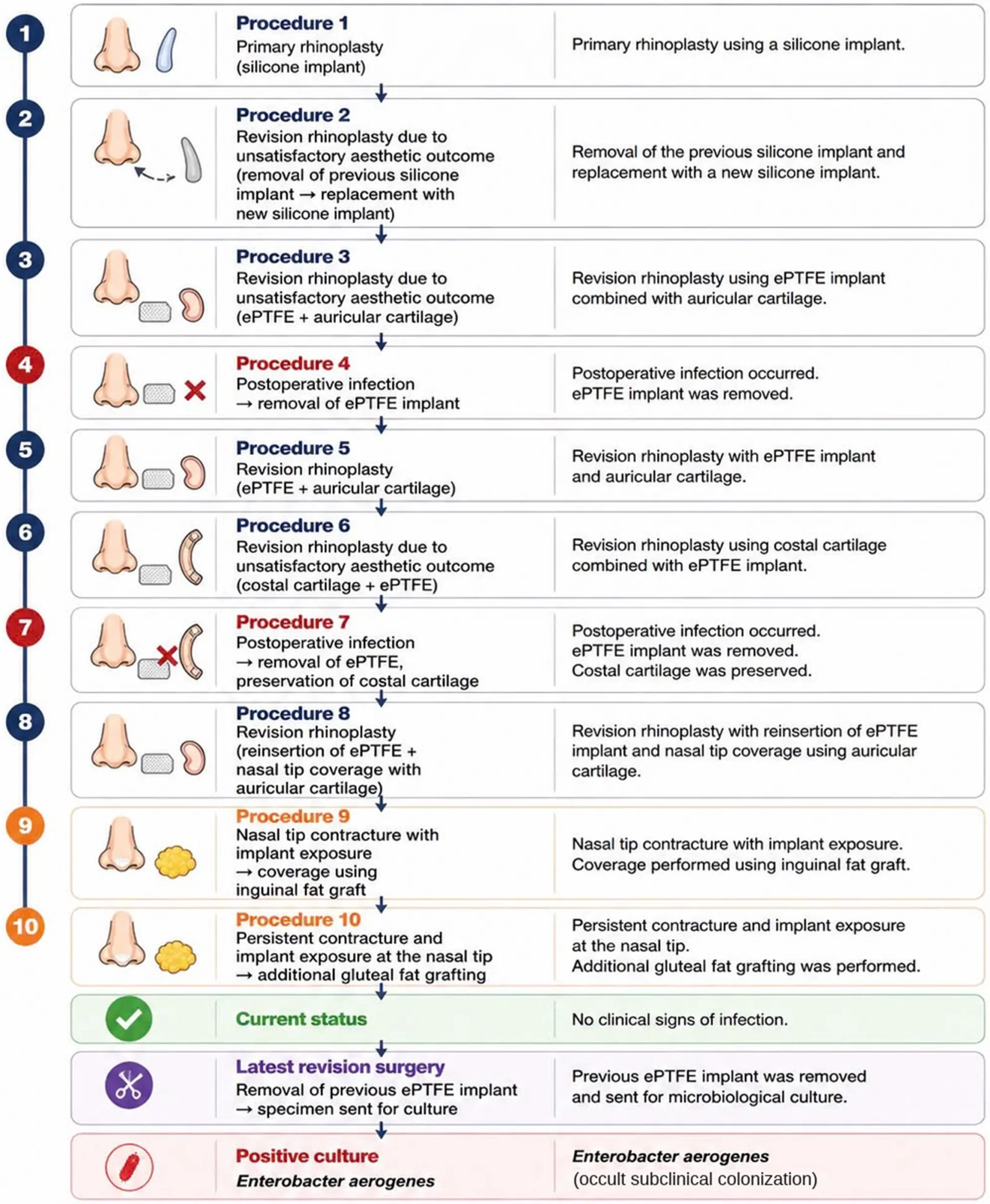
Clinical timeline of the patient’s ten previous rhinoplasty procedures and associated complications (including two postoperative infections and recurrent implant exposure), culminating in the present revision and an incidental positive culture.

The indication for the present (eleventh) revision was persistent nasal-tip contracture with implant exposure and an associated dorsal–tip esthetic deformity uncorrected by the previous soft-tissue procedures; she had no functional (nasal-obstruction) complaint. Her main expectation was durable autologous restoration of nasal shape with definitive removal of the retained implant. The planned procedure was complete explantation of the residual ePTFE and total structural reconstruction with autologous costal cartilage.

She exhibited no signs of infection (swelling, erythema, pain, or discharge), and preoperative laboratory tests (complete blood count, C-reactive protein, and coagulation profile) were within normal limits.

Surgery was performed under general anesthesia via an open approach with standard skin antisepsis. The dorsal ePTFE material and residual auricular cartilage grafts were removed, and intraoperative nasal fluid from the implant pocket was collected at explantation and submitted for culture and antimicrobial susceptibility testing. The nasal framework showed significant structural damage and fibrotic adhesions; no purulence, abscess, or acute inflammation was observed. Total structural reconstruction with autologous costal cartilage was completed as planned (Fig. 2).

**Figure 2.**
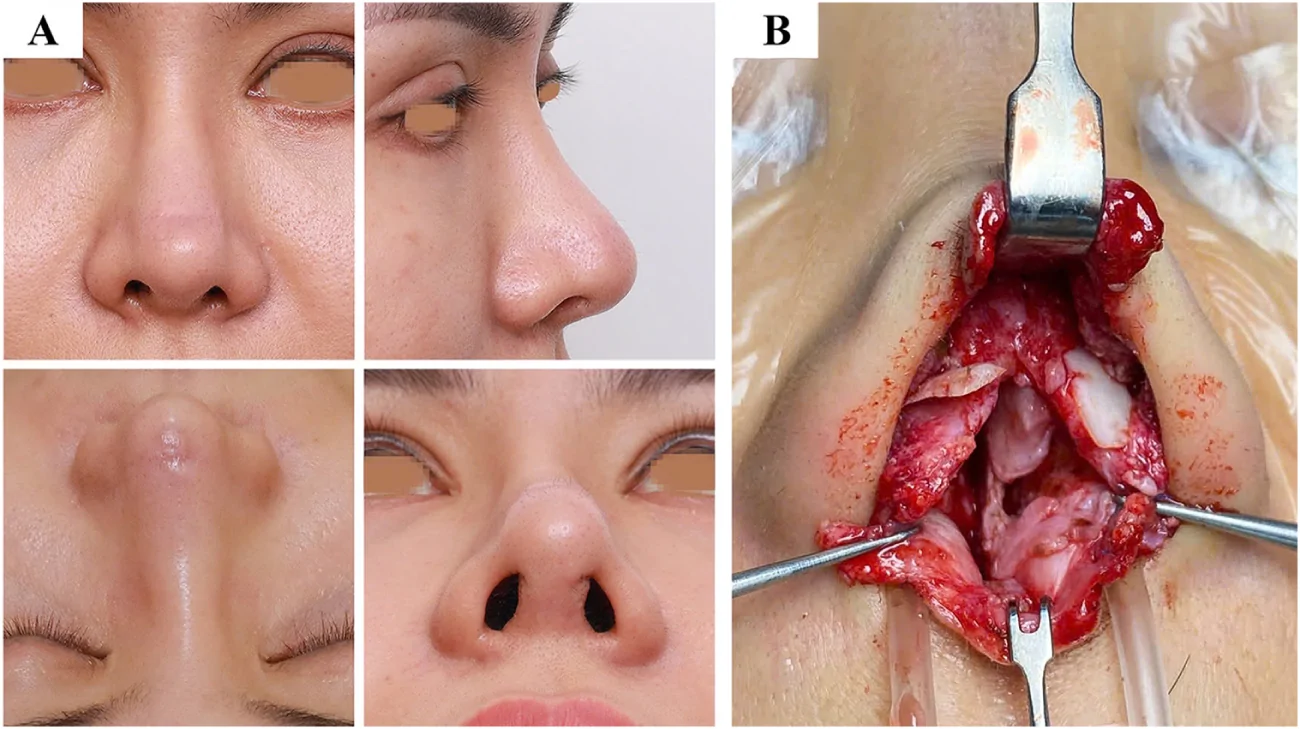
Preoperative nasal photographs and intraoperative findings in revision rhinoplasty. (A) Preoperative nasal photographs after multiple rhinoplasty procedures. (B) Intraoperative exposure of the nasal tip structures and dorsum following the removal of previously implanted material.

Qualitative culture of the intraoperative nasal-fluid specimen yielded *Enterobacter aerogenes*. The isolate was resistant to third-generation cephalosporins [ceftazidime, cefotaxime (MIC ≥ 16 µg/mL), ceftriaxone, and cefoperazone] and to cefuroxime, but susceptible to cefepime, the carbapenems (imipenem, ertapenem, meropenem), aminoglycosides (gentamicin, amikacin), fluoroquinolones [ciprofloxacin, levofloxacin (MIC ≤ 0.5 µg/mL)], the β-lactam/β-lactamase-inhibitor combinations tested, and trimethoprim–sulfamethoxazole (Fig. 3) – a pattern characteristic of an inducible AmpC β-lactamase. Despite the positive culture, the patient had no clinical, laboratory, or intraoperative evidence of infection, consistent with occult colonization of the retained implant rather than active infection.

**Figure 3.**
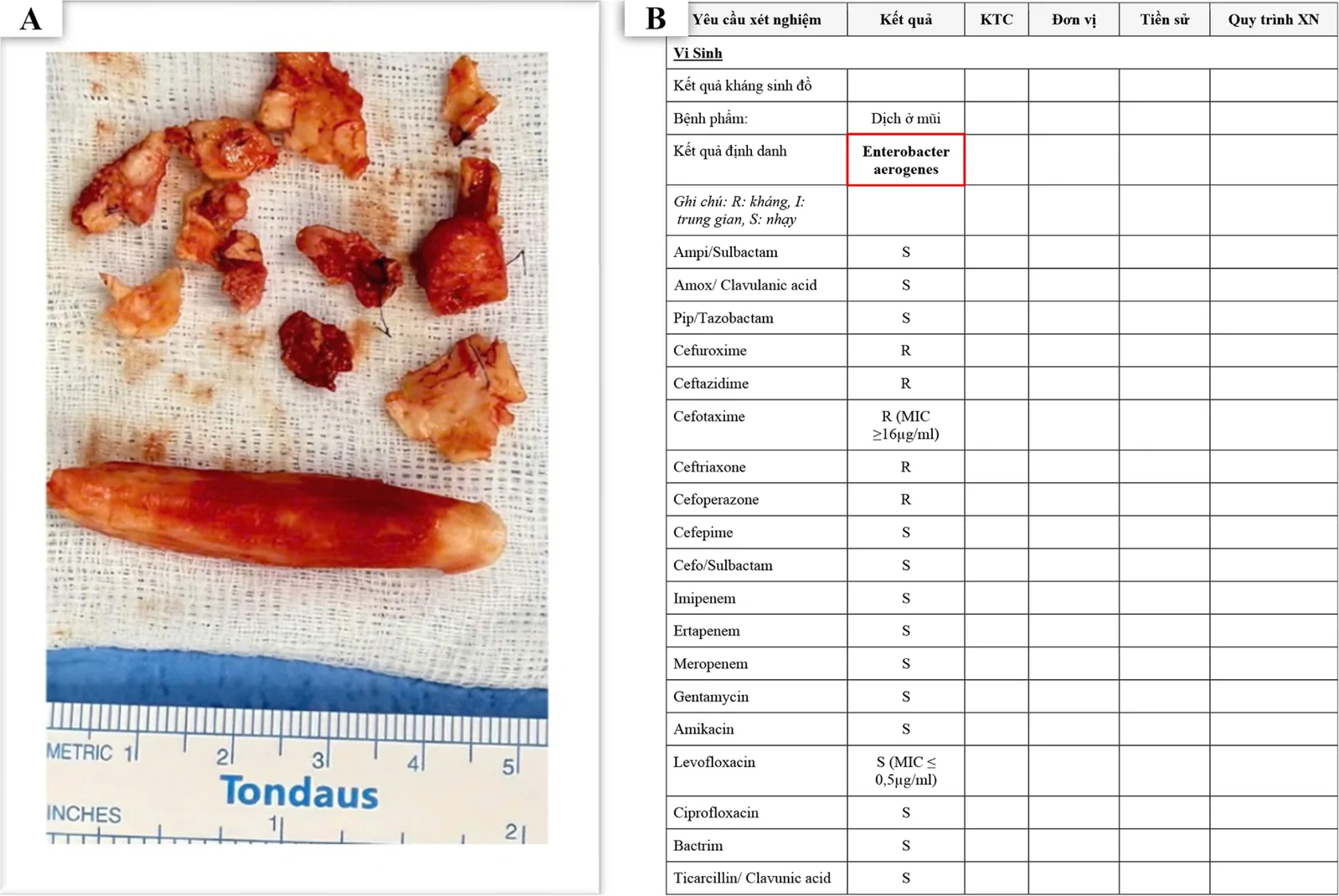
Explanted materials and microbiological findings: (A) Previously implanted ePTFE material and autologous cartilage grafts removed during revision rhinoplasty; (B) Microbiological culture and antimicrobial susceptibility testing of the intraoperative nasal-fluid specimen demonstrated growth of *Enterobacter aerogenes*. The isolate was resistant to third-generation cephalosporins [ceftazidime, cefotaxime (MIC ≥ 16 µg/mL), ceftriaxone, and cefoperazone] and to cefuroxime, while remaining susceptible to cefepime; the carbapenems (imipenem, ertapenem, meropenem); aminoglycosides (gentamicin, amikacin); fluoroquinolones [ciprofloxacin, levofloxacin (MIC ≤ 0.5 µg/mL)]; β-lactam/β-lactamase-inhibitor combinations; and trimethoprim–sulfamethoxazole – an antibiogram characteristic of AmpC β-lactamase production.

Because a viable Gram-negative organism had been confirmed from explanted material adjacent to the newly placed autologous costal cartilage framework – a risk factor for graft colonization and delayed surgical-site infection – a pre-emptive, culture-directed, 7-day course of intravenous meropenem, 1 g every 12 hours, plus amikacin, 500 mg daily, was administered to protect the reconstruction, alongside source control via complete implant removal. The postoperative course was uneventful; at three-month follow-up, the surgical site remained stable, with no surgical-site infection, graft resorption, extrusion, or other complication.

## Discussion

This case illustrates occult bacterial colonization of an ePTFE implant identified during revision rhinoplasty. Despite the absence of clinical signs and normal preoperative laboratory findings, culture of the explanted material grew *Enterobacter aerogenes*, underscoring the silent yet potentially significant nature of latent implant colonization.

Terminology warrants comment. The recovery of viable organisms without a host inflammatory response is, by convention, colonization rather than infection; we therefore describe it as occult or latent colonization. The distinction is not merely semantic: biofilm-embedded organisms can remain clinically silent yet act as a reservoir for later infection, particularly when tissue integrity is compromised or new grafts are introduced. In the setting of biofilm, however, the boundary between colonization and subclinical infection is not absolute, and longitudinal data would be required to determine whether the organisms recovered here would ultimately have progressed to overt infection.

Infection rates in primary rhinoplasty are low (approximately 3%)[4] but rise markedly after multiple revisions and prior implant use, owing to scarring, reduced vascularity, altered anatomy, and foreign material; Tran and Jang reported infection rates roughly 19-fold higher in revision than in primary rhinoplasty (3.63% vs. 0.19%)[5]. A positive culture in a patient with ten prior procedures is therefore not, in isolation, unexpected, and we claim no novelty on that basis. The distinctive contribution lies in the combination of truly occult colonization (no clinical, laboratory, or intraoperative signs), microbiological confirmation of an atypical Gram-negative organism, and incidental detection on explanted ePTFE – supporting a specific recommendation for routine culture of explanted material rather than merely reaffirming the known elevated risk of revision surgery.

ePTFE’s microporous structure permits fibrovascular ingrowth that improves stability but may also facilitate bacterial adhesion and biofilm formation[6]. Biofilms embed bacteria in an extracellular matrix, conferring resistance to antibiotics and host defenses[1], so eradication often requires implant removal[6]. Biofilms may also harbor persister cells – dormant bacteria that survive antibiotic exposure and contribute to recurrence^[^1,7^]^ – and biofilm and persister-cell formation have been demonstrated in revision rhinoplasty, even in the absence of implants[8]. These mechanisms plausibly explain the persistence of viable organisms on the explanted material despite the absence of clinical infection.

The most common rhinoplasty pathogens are Gram-positive skin flora such as *Staphylococcus aureus* and *S. epidermidis*, although Gram-negative opportunists may occur[9]. *Enterobacter aerogenes* (recently reclassified as *Klebsiella aerogenes*) is rarely reported in rhinoplasty; Jiang et al. recovered it in 10 of 123 post-rhinoplasty infection samples, supporting its plausibility in implant-associated colonization[10]. Our isolate showed the classic AmpC β-lactamase phenotype – resistance to third-generation cephalosporins with preserved cefepime and carbapenem susceptibility – consistent with the chromosomal, inducible AmpC intrinsic to this organism, which can be de-repressed to confer clinically significant cephalosporin resistance. Because AmpC may be amplified during cephalosporin therapy, a carbapenem-based regimen is preferred for serious AmpC-producing *Enterobacterales*; this, along with the documented carbapenem susceptibility, guided our antibiotic choice[11].

The principal strength of this report is the microbiological confirmation, with full susceptibility profiling, of Gram-negative colonization on an explanted implant in a completely asymptomatic patient – a finding of direct relevance to revision rhinoplasty. Several limitations should nevertheless be acknowledged. Culture was qualitative rather than quantitative, as colony counts are not standard for explant specimens and lack a defined significance threshold in this setting; biofilm and persister-cell involvement was inferred from the clinical, microbiological, and published context rather than demonstrated directly by sonication or electron microscopy; and, as a single case, causal and prognostic inferences – including the untreated natural history of the colonization – remain limited.

## Conclusion

Bacteria may persist as latent colonizers on implanted materials in rhinoplasty for prolonged periods without producing overt clinical infection. This case report highlights the possibility of bacterial colonization of implants even in the absence of clinical, laboratory, and intraoperative signs of infection, and the need for culture-directed antibiotic use – particularly when explanting retained materials during revision rhinoplasty in patients with multiple prior procedures.

## Data Availability

All data supporting the findings of this case report are included within the article. Further details are available from the corresponding author on reasonable request, subject to patient confidentiality.

## Previous presentation/concurrent submission

This case has not been presented at a conference and is not under consideration by any other journal.
